# Pharmacy Students' Knowledge Assessment of* Naegleria fowleri* Infection

**DOI:** 10.1155/2016/2498283

**Published:** 2016-02-15

**Authors:** Sadia Shakeel, Wajiha Iffat, Madeeha Khan

**Affiliations:** ^1^Dow College of Pharmacy, Dow University of Health Sciences, Karachi, Sindh 75270, Pakistan; ^2^Faculty of Pharmacy, University of Karachi, Karachi, Sindh 75270, Pakistan

## Abstract

A cross-sectional study was conducted from April to August 2015 to assess the knowledge of pharmacy students towards* Naegleria fowleri* infection. A questionnaire was distributed to senior pharmacy students in different private and public sector universities of Karachi. Descriptive statistics were used to demonstrate students' demographic information and their responses to the questionnaire. Pearson chi-square test was adopted to assess the relationship between independent variables and responses of students. The study revealed that pharmacy students were having adequate awareness of* Naegleria fowleri* infection and considered it as a serious health issue that necessitates instantaneous steps by the government to prevent the general public from the fatal neurological infection. The students recommended that appropriate methods should be projected in the community from time to time that increases public awareness about the associated risk factors.

## 1. Introduction

**Figure 1:**
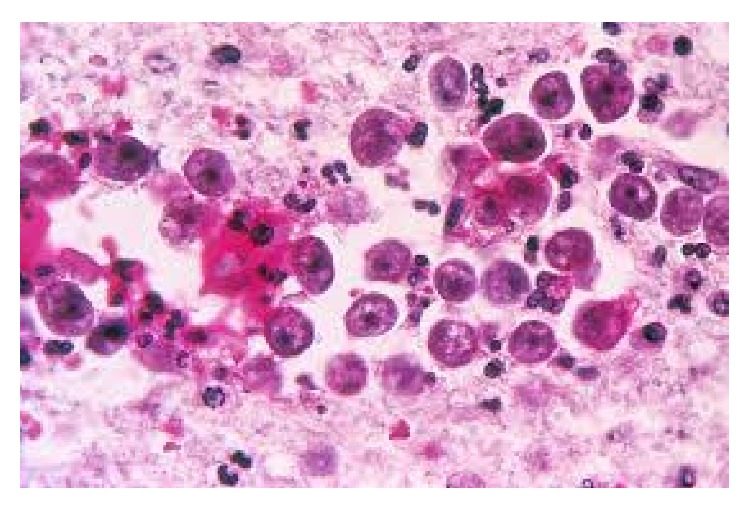
Microscopic view of* Naegleria fowleri*.

*Naegleria fowleri (N. fowleri)* is generally found as an amoeba or as a free flagellum in warm lakes, hot springs, and fresh water reservoirs including rivers, ponds, and unchlorinated swimming pools (Figure 1). As* N. fowleri* is a heat tolerant (thermophilic) protist, it thrives throughout summer when temperatures are elevated [1]. The organism gains access to the human brain through the nostrils while washing face, swimming, or performing ritual ablution. It then pierces the cribriform plate to enter the central nervous system where it causes granulomatous inflammation leading to primary amoebic meningoencephalitis (PAM) [2]. PAM due to* N. fowleri* is an acute, fulminant, necrotizing, hemorrhagic meningoencephalitis, characterised by severe headache, stiff neck, fever (38.5°C–41°C), altered mental state, seizures, and coma. It is almost always fatal within an average of 3–7 days [3]. PAM does not have effective therapeutic options and is a rapidly fatal infection. The time period between initial contact with the pathogenic* N. fowleri* and the onset of clinical signs and symptoms varies from 2 to 3 days up to as long as 7–15 days depending partly on the size of the inoculum and the virulence of the strain. The significance and enormity of this amoebic infection can be explained in terms of its alarming mortality rate. Nearly about 200 cases of PAM have been reported throughout the world. Recently, 13 cases of this rare life-threatening infection were reported from the coastal city of Karachi, Pakistan [1].

Pakistan, and particularly Karachi, being a subtropical region, predominantly has a warm climate providing an encouraging ecological niche for this organism to occupy. The first case of* N. fowleri *was reported in Pakistan in 2008. Since then, number of cases has been reported and its ever increasing occurrence rate over the years is making it particularly worrisome. In 2011, 13 cases of this rare life-threatening infection were reported from Karachi [1]. In 2014, one more fatal case was noted [4]. These cases have thrown light on various factors leading to progression of this infection and resultant high mortality.

Pakistan is a Muslim country, where majority of the people practice nasal irrigation as part of ablution. Being a developing state, with a high rate of illiteracy and poverty, the health systems facilities are still nonexistent and unreachable in many parts of the country. Lack of potable water supply and proper sanitation, with lack of awareness, further adds to such problems of epidemics of water borne diseases. Therefore, it is likely that cases of* N. fowleri *infections will continue to peak. Public awareness regarding this disease, along with proper treatment of water, either by chlorination or boiling, should be carried out to ensure that the risk of infection declines. The authorities responsible should identify the health problems of masses and take concrete measures to salvage the misery. With this background, the present study was conducted to evaluate the knowledge of pharmacy students towards* N. fowleri *infection.

## 2. Materials and Methods

This cross-sectional study was conducted from April to August 2015 through adoption of a questionnaire distributed to senior pharmacy students (fourth and final year) in different private and public sector universities of Karachi. In total, 314 pharmacy students participated in the study. The questionnaire was developed to acquire students' demographic data and their knowledge about* N. fowleri* infection. The questionnaire items were analyzed with SPSS 20.0 software. Descriptive statistics were used to demonstrate students' demographic information and their responses. Pearson chi-square test was executed for evaluating the relationship between gender, institution, and professional year of students with their responses. A value of *p* < 0.05 was considered significant.

## 3. Results

Out of 450 survey questionnaires, only 314 were returned back (response rate was 69.77%).  Table 1 shows the demographic characteristics of study population that includes 89.81% and 10.19% females and males, respectively. Nearly 60% belonged to public and 40.5% to private sector universities. Over 50% of the participants were final year students.

**Figure 2:**
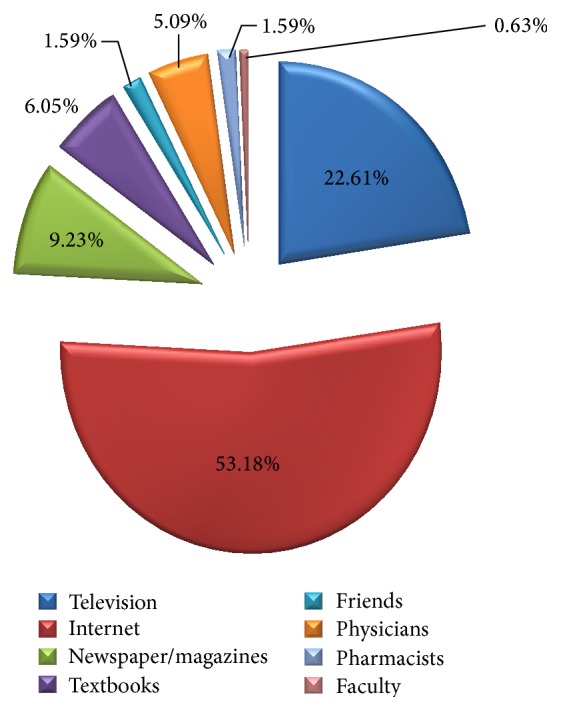
Students' perspectives regarding the best source of information for health news.

Inquiring the knowledge of pharmacy students about* N. fowleri* infection, 55.1% considered themselves to be somewhat knowledgeable. Internet (53.18%) was considered to be the best source of information for health news. (Figure 2) Television (22.61%) and newspaper (9.23%) were the next important sources of information from students' perspectives. The majority (64.6%) knew that amoeba is the causative agent of* N. fowleri* infection whereas 25.8% considered virus to be the major cause.

The general perspectives of students regarding* N. fowleri* infection are summarized in Table 2. Around 95% knew that central nervous system (CNS) is typically affected by an infection. Mass population (93%) considered infection to be preventable. Around 70% of students were acquainted with the fact that* N. fowleri* infection most commonly occurs in summer. More than 80% considered it true that* N. fowleri* infects people when water containing amoeba enters the body through the nose. More than 90% negated that infection can spread from one person to another and agreed that infection travels to the brain where it destroys brain tissue. On inquiring about sign or symptom of* N. fowleri* infection, the responses included fever (69.4%), severe headache (72.3%), stiff neck (39.2%), nausea and vomiting (54.1%), confusion (35.7%), loss of balance (27.4%), sleepiness (25.8%), seizures (31.5%), and hallucinations (18.8%).

**Figure 3:**
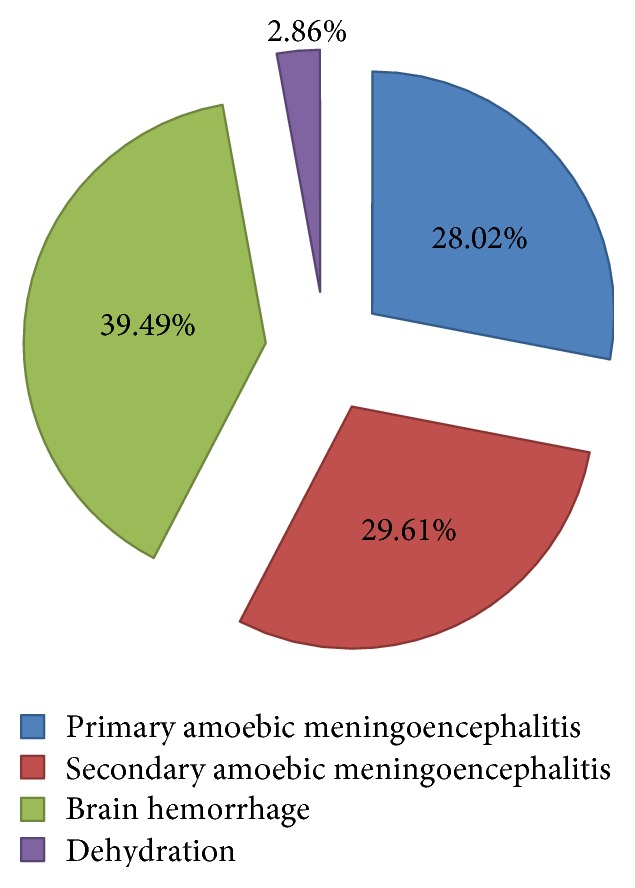
Students' perspectives regarding the cause of death in* N. fowleri* infection.

Table 3 depicted the responses of students regarding protective measures against infection. A considerable percentage of students knew that standard chlorination of swimming pools, use of nose clips when taking part in water-related activities, and avoiding water-related activities in warm freshwater during periods of high water temperature can be helpful in preventing from infection. According to responses of students, patients who get an infection are most likely to die because of primary amoebic meningoencephalitis (28.02%), secondary amoebic meningoencephalitis (29.61%), brain hemorrhage (39.49%), and dehydration (2.86%) (Figure 3).

Around 67% considered that the initial signs and symptoms of infection begin in 1–7 days whereas 37.3% believed that, after the onset of symptoms, the disease progresses rapidly and usually results in death in 5–14 days. Nearly half of the population (48.4%) did not know whether* N. fowleri* infection can be cured or not. Pearson chi-square test was executed for evaluating the relationship between gender, institution, and professional year of students with their response. Significant association was found between the professional year of students and majority of their responses (*p* value < 0.05). However, no significant association exists between gender and institution of students and responses.

## 4. Discussion

*N. Fowleri* is the only species of its genus* Naegleria* which causes fatal infections to human brain. Owing to the rarity of infection and complexity in early recognition, about 75% of diagnoses are made following the death of patient.* N. fowleri* is not regarded as an opportunistic pathogen since it normally resides in persons. Indeed, PAM often prevails in immunologically strong children and adults during recreation in warm bodies of freshwater [5]. In this study, mass population (93%) considered that the infection is preventable. Students (43% and 28.7%) considered that adults and teenagers group has the highest reported rate of* N. fowleri* infection, respectively.

Community gets infected with* N. fowleri* if water having the amoeba goes into the body through nose and typically happens while swimming or diving in warm freshwater places.* N. fowleri* amoeba then travels up the nose to the brain and destroys brain tissue. Research showed that infection cannot be initiated by drinking contaminated water [6]. In rare cases, infection can also occur when contaminated water from other sources (such as inadequately chlorinated swimming pool water or contaminated tap water) enters the nose, for instance, if people dip their heads or clean their noses in religious practices [7, 8]. Our research also revealed the similar knowledge of students towards the occurrence of* N. fowleri* infection. In the current study, around 67% considered that the initial signs and symptoms of infection begin in 1–7 days whereas 37.3% believed that, after the onset of symptoms, the disease progresses rapidly and usually results in death in 5–14 days. However, research has shown that the time from initial contact to the onset of illness is approximately 5–8 days but may be as short as 24 hours. The remarkable attribute of PAM is the speedy onset of symptoms subsequent to exposure. The disease progresses quickly, and, with no timely diagnosis and intervention, death typically occurs in a week or less [9]. In our study, 70% of students were acquainted with the fact that* N. fowleri* infection most commonly occurs in summer.* N. fowleri* is a thermophilic amoeba and consequently proliferates in water when the ambient temperature increases above 30°C. With the predictable temperature increase ensuing from global warming, it is likely that cases of* N. fowleri* PAM may be seen even in countries where it had previously not been reported [10]. Some of the initial symptoms include bifrontal or bitemporal headaches not responsive to analgesics and fevers ranging from 38 to 41°C [11]. A variation in taste or smell or even rhinitis can be apparent early [12]. Photophobia may occur shortly in the clinical course and may be followed by neurological abnormalities such as lethargy, confusion, seizures, coma, diplopia, or bizarre behavior [9]. On inquiring about the sign or symptom of infection in our study, the responses included fever (69.4%), severe headache (72.3%), stiff neck (39.2%), nausea and vomiting (54.1%), confusion (35.7%), loss of balance (27.4%), sleepiness (25.8%), seizures (31.5%), and hallucinations (18.8%). The infection destroys brain tissue causing brain swelling and death [5]. According to responses of students, patients who get an infection are most likely to die because of primary amoebic meningoencephalitis (28.02%), secondary amoebic meningoencephalitis (29.61%), brain hemorrhage (39.49%), and dehydration (2.86%).

Our students (69.43%) considered that standard chlorination of swimming pools can prevent the spread and/or acquisition of an infection. Amoeba proliferation can be restricted by satisfactory chlorination of heavily used swimming pools, particularly during summer months as* N. fowleri* is susceptible to chlorine in water (one part per million) [7]. Local public health authorities should monitor recreational waters for* N. fowleri* amoebae and appropriate warnings should be posted, particularly in high-risk areas. A number of drugs have been found to be effective against* N. fowleri in vitro*. Though, their usefulness is uncertain in view of the fact that nearly all infections have been lethal, even when people were treated with analogous drug combinations [3]. There are only four recognized survivors in North America: one from the USA in 1978, one from Mexico in 2003, and two from the USA in 2013 [13]. In the current study, nearly half of the population (48.4%) did not know whether* N. fowleri* infection can be cured or not.

## 5. Conclusions

It is concluded that pharmacy students were having adequate awareness of* N. fowleri* infection and considered it as a serious health issue that necessitates instantaneous steps by the government to prevent the general public from this fatal neurological infection. Health authorities should adopt such policies that assure prevention of this disease. One major step is to make sure that the water supply to the city should be chlorinated according to suggested standards. The students recommended that appropriate methods for prevention should also be projected in the community from time to time that increase public awareness about the associated risk factors.

## Figures and Tables

**Table 1:** Characteristics of study population.

Characteristics	Percentages
Gender	
Male	10.19%
Female	89.81%
Academic year	
Forth year	48.32%
Fifth year	51.68%
Institute	
Public sector	59.5%
Private sector	40.5%

**Table 2:** Perspectives of students regarding *N. fowleri* infection.

Statement	Correct response	Incorrect response
*N. fowleri* infects people when water containing amoeba enters the body through nose	80.25%	19.74%
It typically occurs when people go swimming or diving in warm freshwater places	61.46%	38.54%
*N. fowleri* travels up the nose to brain where it destroys brain tissue	90.13%	9.87%
One can be infected with *N. fowleri* by drinking contaminated water	80.89%	19.11%
*N. fowleri* can survive in properly chlorinated poolsor salt water	81.21%	18.79%
*N. fowleri* is harmful if swallowed	82.17%	17.83%
*N. fowleri* is present in distilled, sterile, or previously boiled water	89.17%	10.83%
An infection can spread from one person to another	92.04%	7.96%

**Table 3:** Responses of students regarding the protective measures.

Statement	Correct response	Incorrect response
Standard chlorination of swimming pools	69.43%	30.57%
Covering mouth when coughing or sneezing	82.48%	17.52%
Avoiding aspiration of freshwater into the nose	58.92%	41.08%
Use of vitamins and herbal supplements	97.77%	2.23%
Using antivirals/antibiotics	92.36%	7.64%
Staying home and avoiding public places	97.45%	2.55%
Wearing protective equipment in public places	82.17%	17.83%
Avoiding swimming in or jumping into warm freshwater lakes and rivers	47.77%	52.23%
Using nose clips when jumping or diving into warm bodies of fresh water	57.96%	42.04%
